# Socioeconomic inequalities in patients undergoing abdominal wall reconstruction in the North-West of England, UK: a three-centre retrospective cohort study

**DOI:** 10.1007/s10029-024-03155-0

**Published:** 2024-09-13

**Authors:** Donna Shrestha, Theodoros M. Bampouras, Clifford L. Shelton, Dominic Slade, Daren A. Subar, Christopher J. Gaffney

**Affiliations:** 1 Lancaster Medical School, Lancaster University, Lancaster, LA1 4AT UK; 2 Blackburn Research Innovation Development Group in General Surgery (BRIDGES), Blackburn, UK; 3 School of Sport and Exercise Sciences, Liverpool John Moores University, Liverpool, UK; 4 Department of Anaesthesia, Wythenshawe Hospital, Manchester University NHS Foundation Trust, Manchester, UK; 5 Department of General Surgery, Salford Royal Hospital, Manchester, UK; 6 Department of General Surgery, East Lancashire Hospitals NHS Trust, Blackburn, UK

**Keywords:** Abdominal Wall Reconstruction, Ventral hernia repair, Hernia, Surgical outcomes, Health inequalities, Socioeconomic status

## Abstract

**Purpose:**

Patients from deprived areas are more likely to experience longer waiting times for elective surgery, be multimorbid, and have inferior outcomes from elective and emergency surgery. This study aims to investigate how surgical outcomes vary by deprivation for patients undergoing elective abdominal wall reconstruction.

**Methods:**

A three-centre retrospective cohort study was conducted across three hospitals in North-West England, including patients with complex ventral hernias undergoing abdominal wall reconstruction between 2013 and 2021. Demographic data, comorbidities, and index of multiple deprivation quintiles were recorded.

**Results:**

234 patients (49.6% female), age 57 (SD 13) years, underwent elective abdominal wall reconstruction. Significantly higher unemployment rates were found in the most deprived quintiles (Q1 and Q2). There were more smokers in Q1 and Q2, but no significant deprivation related differences in BMI, diabetes, chronic kidney disease or ischaemic heart disease. There were also higher rates of Clavien-Dindo 1–2 complications in Q1 and Q5, but no difference in the Clavien-Dindo 3–4 outcomes. Patients in Q1 and Q5 had a significantly greater hospital length of stay.

**Conclusion:**

The association between deprivation and greater unemployment and smoking rates highlights the potential need for equitable support in patient optimisation. The lack of differences in patient co-morbidities and hernia characteristics could represent the application of standardised operative criteria and thresholds. Further research is needed to better understand the relationship between socioeconomic status, complications, and prolonged hospital length of stay.

**Supplementary Information:**

The online version contains supplementary material available at 10.1007/s10029-024-03155-0.

## Introduction

It is estimated that 10.8 million general surgical procedures are performed annually in England, UK [1] and approximately 15,000 hospital admissions are attributed to incisional hernia surgery [2]. Abdominal wall reconstruction (AWR) surgery addresses the most complex of incisional hernias, with greater patient co-morbidities and technical demands [3]. The need to understand socioeconomic inequalities in patients undergoing abdominal wall reconstruction (AWR) is paramount because AWR is developing as a distinct surgical sub-specialty and there are increasingly prominent calls to improve care for the management of hernias, which has been historically heterogenous and variable in quality [4–7]. Understanding individual patients’ needs and their socioeconomic challenges is crucial for informing equitable care. It has been projected that optimal patient care at every stage of the AWR patient journey could lead to savings of £20,000 per patient [2]. Efforts to standardise care offers potential financial benefits to healthcare systems such as the National Health Service (NHS) but also offers potential to improve post-operative outcomes for patients and their quality of life.

Health inequalities, the avoidable differences in health outcomes between specific groups of people, have increased in recent years in the UK and the health gap between the least and most deprived populations has widened [8]. Patients from the most deprived areas are over twice as likely to wait more than a year for elective surgery, compared to those from the least deprived areas [9]. Furthermore, there is growing evidence of disparities in surgical outcomes in many areas of surgery related to socioeconomic deprivation [10–13]. Data from the national emergency laparotomy audit (NELA) database has demonstrated that patients from deprived backgrounds have greater 30-day mortality and more co-morbid [10]. Socioeconomic deprivation has been linked with higher peri-operative and long term mortality rates in elective colorectal cancer surgery [11], as well as lower survival rates following endometrial cancer surgery, regardless of the stage of the disease at diagnosis [14]. A comprehensive cross-specialty study involving over 9,000 patients who underwent elective surgery demonstrated that those from more deprived backgrounds were subject to greater long-term complications [12]. The published literature on socioeconomic inequalities on ventral hernia surgery outcomes originates primarily from the USA and, most commonly, use insurance status and median household income as indicators of socioeconomic status [15–20]. Although research in the field of socioeconomic inequalities and health outcomes is rapidly emerging, to our knowledge, there are no published studies based on UK data on the socioeconomic inequalities in ventral hernia surgery outcomes.

Rates of incisional hernias following midline laparotomy have been reported to be up to 40%, depending on the period of follow up [21–23]. The development of incisional hernias is influenced by multiple factors, including obesity, smoking, comorbidities, and malnutrition, all of which hinder wound healing [24]. In the North-West of England, where there are greater levels of socioeconomic deprivation compared to other areas in the country, there is a higher incidence of cardiometabolic diseases such as hypertension, diabetes, obesity and ischaemic heart disease [25]. The pathways in which socioeconomic deprivation could adversely impact outcomes in patients undergoing AWR is multifaceted and understudied. However, socioeconomic deprivation could manifest adversely through known theorised mechanisms such as delayed health care utilisation [26], poorer health-seeking behaviours and health literacy [27, 28], greater multimorbidity and lifestyle related risk factors [29, 30]. The aim of this study is to (i) understand how patient factors vary by socioeconomic deprivation, and (ii) determine whether patients from more deprived backgrounds have greater adverse outcomes of AWR surgery.

## Methods

### Study design and ethics

This was a retrospective, three-centre observational cohort study that was performed through the analysis of retrospectively collected data from electronic patient records. The study sites were hospitals where complex ventral hernia surgeries are routinely performed and included Northern Care Alliance NHS Foundation Trust, University Hospitals of Morecambe Bay NHS Foundation Trust, and East Lancashire Hospitals NHS Trust. Ethical approval was granted by Lancaster University (FHM-2022-3281-IRAS-1) and Health Research Authority (HRA) approval was granted before the research commenced.

### Subjects and inclusion criteria

The inclusion criteria comprised: adults aged ≥ 18 years, with complex primary or incisional hernias where ‘complex’ was defined as hernias with large defect ≥ 10 cm, or previous repair, or previous mesh, or need for component separation, or need for adhesiolysis [3, 31]. All patients had a minimum of two years of follow up. Patients undergoing parastomal hernia repairs were excluded due to the unique challenges that parastomal repairs pose.

Patients’ socioeconomic status was derived from the English Index of Multiple Deprivation (IMD) decile, which was obtained from individual postcodes. The IMD is a composite score made of scores across seven deprivation domains (income, employment, health and disability, education skills and training, crime, barriers to housing and services, and living environment) with a total of 37 indicators, where each domain measures the proportion of the population experiencing a certain category of deprivation. 32,844 Lower-layer Super Output Areas (LSOA) in England are ranked from most deprived (1) to the least deprived (32,844) based on their IMD score. Each LSOA accounts for an average of 1500 residents [32]. For the purposes of this analysis, patients were grouped into deprivation quintiles (Q1, most deprived - Q5, least deprived).

### Statistics

Data were assessed for normality using the Shapiro-Wilk test. A p-value of *p* < 0.05 was considered a significant deviation from normality. Normality was also defined as the ratio of skewness and kurtosis to the respective standard error not exceeding ± 2.0.

Baseline patient characteristics, hernia characteristics and adverse outcomes were compared between IMD quintiles (Q1-Q5) using descriptive statistics. For continuous variables with a normal distribution, a one-way analysis of variance (ANOVA) to examine for differences was used. Kruskal-Wallis test was used for non-parametric variables. For categorical variables, the chi-squared goodness of fit test was used. A p-value of *p* < 0.05 was considered significant for all tests.

Two binomial multivariable logistic regression were performed to assess the relationship between deprivation and adverse outcomes and test the hypothesis that low socioeconomic status predicts poor outcomes of surgery: (i) to ascertain the effects of age, sex, BMI, diabetes, smoking status, chronic lung disease, ischaemic heart disease, the number of previous repairs ≥ 1, previous mesh, component separation, having an open procedure and IMD on the likelihood of having a prolonged LOS, and (ii) to ascertain the effects of age, sex, BMI, diabetes, smoking status, chronic lung disease, ischaemic heart disease, component separation, having an open procedure, CDC wound classification and IMD on the likelihood of having post operative complications. Results were presented as odds ratios (OR) to represent the effect size of predictor variables on the dependent variable. 95% confidence intervals were also calculated. Variables were checked for multicollinearity using the variance inflation factor (VIF) and were not included if the value was ≥ 1.5. A comprehensive set of assumption checks were performed to ensure the validity and reliability of the model. There were no indications of significant multicollinearity (VIF < 5, tolerance > 0.5) and Cook’s Distance values were within acceptable ranges (-2.5 to 2.5), confirming that no data transformation was required. All analyses were conducted using Jamovi (version 2.4.8, The Jamovi Project, Sydney, Australia).

## Results

### Baseline characteristics

A total of 234 patients underwent an elective complex ventral hernia repair between 2013 and 2021. The percentage of patients in each quintile, Q1-Q5, were 39.3%, 20.5%, 9.8%, 17.9% and 12.5% respectively and differed significantly (*p* < 0.001). The mean patient age was 56.9 (SD, 13.2) years; 111 (49.6%) patients were female (Table 1). There was no difference in age (*p* = 0.22), sex (*p* = 0.109), or BMI (*p* = 0.058) between the five deprivation quintiles. Employment status varied across the five deprivation quintiles (*p* < 0.001), with unemployment rates being higher in Q1 and Q2, i.e. the most deprived quintiles. There was a significant difference in current smokers across the quintiles (*p* = 0.03, with a greater proportion of current smokers in Q1 and Q2. No significant differences were observed between the five quintiles in the presence of diabetes.

**Table 1:** Baseline characteristics comparison by IMD quintile. Percentages displayed are of the overall total or relative to the total number in each IMD quintile, as indicated by n/total. Chi squared goodness of fit or ANOVA tests were used. P-values in bold are significant (*p* < 0.05)

	Missing data	Quintile 1 (Q1)	Quintile 2 (Q2)	Quintile 3 (Q3)	Quintile 4 (Q4)	Quintile 5 (Q5)	*P*-value
**Total ** *n * **(%)**	0	88/224 (39.3)	46/224 (20.5)	22/224 (9.8)	40/224 (17.9)	28/224 (12.5)	**< 0.001**
**Age mean (SD)**	0	55 (14)	59 (13)	54 (13)	60 (12)	59 (14)	0.122
**Sex**							
Sex - F *n* (%)	0	48/88 (54.5)	25/46 (54.3)	12/22 (54.5)	13/40 (32.5)	13/28 (46.4)	0.109
**BMI mean (SD)**	3	31.1 (5.8)	31.2 (7.1)	34.5 (7.3)	29.9 (4.4)	29.6 (4.4)	0.058
**Employment** **Status n**	36	74	37	18	38	21	
Unemployed *n* (%)		29/74 (39.2)	16/37 (43.2)	2/18 (11.1)	7/38 (18.4)	4/21 (19.0)	**< 0.001**
Retired *n* (%)		17/74 (22.9)	8/37 (21.6)	1/18 (5.5)	10/38 (26.3)	7/21 (33.3)	**< 0.001**
**Smoking Status ** *n*	2	87	46	22	39	28	
Current *n* (%)		17/87 (19.5)	8/46 (17.4)	4/22 (18.2)	3/39 (7.7)	2/28 (7.1)	**0.030**
**Diabetes ** *n * **(%)**	0	14/88 (15.9)	9/46 (19.6)	3/22 (13.6)	4/40 (10)	2/28 (7.1)	0.124

### Hernia characteristics

A total of 218 (97.3%) of patients had incisional hernias and the remaining six (2.7%) patients had primary ventral hernia repairs. 149 (66.5%) patients underwent their first hernia repair. Whereas 80 (35.7%) patients had one or more previous repairs (ranging from one to five repairs). 64 (28.6%) patients had a mesh inserted at a previous repair. The mean cranio-caudal hernia size was 12.8 cm (SD, 7.8) and the mean transverse hernia size was 10.9 cm (SD, 5.4). The most common ventral hernia working group classification [33] was grade 2 (55.4%), followed by grade 1 (19.6%), grade 3 (15.6%) and grade 4 (8.9%). There was no difference in the number of previous repairs that patients had in each of the five deprivation quintiles, with more than 60% of each group undergoing their first repairs. (Table available in supplement 2)

### Operative details

There were six different operating surgeons across three hospital sites. 191 (85.6%) of patients had an open repair and 32 (14.4%) had laparoscopic or laparoscopic-assisted procedures. 42.4% of repairs underwent component separation (Table 2).

**Table 2:** Operative details

	*n*	%
**Incisional hernia**	218	97.3
**Primary hernia**	6	2.7
**Laparoscopic/** **Laparoscopic-assisted**	32 / 223	14.4
**Open**	191/ 223	85.6
**Component separation**	95 / 224	42.4

### Surgical outcomes

The median length of stay (LOS) was 6 days and 106 (47.3%) patients had a prolonged LOS (greater than the median LOS). Complications occurred in 76 (33.9%) patients. Of these, 58 (25.9%) were Clavien-Dindo (CD) 1–2 complications and 18 (8%) were CD 3–4 complications (CD classification in supplement 1). There was a significant difference in CD 1–2 outcomes amongst the five IMD quintiles (*p* = 0.014) but no difference in the CD 3–4 outcomes (p-0.518) (Table 3). There was a significant difference in prolonged length of stay amongst the groups (*p* = 0.026).

**Table 3:** Adverse outcomes compared by IMD quintiles: prolonged length of stay, Clavien-Dindo (CD) 1 or 2 and CD3 or 4 complications. Percentages displayed are out of the total number in each IMD quintile, as indicated by n/total. *P*-values in bold are significant (*p* < 0.05)

	Quintile 1 (Q1)	Quintile 2 (Q2)	Quintile 3 (Q3)	Quintile 4 (Q4)	Quintile 5 (Q5)	*P* -value
**Prolonged length of stay (> median length of stay)**	47/88 (53.4)	20/46 (43.5)	7/22 (31.8)	14/40 (35)	16/28 (57.1)	**0.026**
**Complications** **CD 1 or 2 (n**,** (%))**	29/88 (33.0)	6/46 (13.0)	4/22 (18.2)	10/40 (25.0)	9/28 (32.1)	**0.014**
**Complications** **CD 3 or 4 (n**,** (%))**	7/88 (8.0)	3/46 (6.5)	1/22 (4.5)	4/40 (10.0)	3/28 (10.7)	0.518

### Logistic regression – prolonged hospital length of stay (Table 4)

The logistic regression model was statistically significant, χ^2^(15) = 76.6, *p* < 0.001. The model explained 39.7% (Nagelkerke R^2^) of the variance in prolonged length of stay and correctly classified 76.5% of cases. Of the variables included in the model, only having component separation was a significant predictor of prolonged hospital length of stay (OR 5.78, *p* < 0.001). Deprivation had no significant impact on the likelihood of having prolonged hospital length of stay (*p* > 0.05).

**Table 4:** Predictive variables for prolonged length of stay assessed using binomial multivariable logistic regression. P-values in bold are significant (*p* < 0.05)

	Odds Ratio	*P*-value	Lower CI	Upper CI
**Age**	1.00	0.78	0.98	1.03
**Sex**:				
Female – Male	1.02	0.96	0.52	2.00
**BMI**	0.95	0.08	0.90	1.01
**Smoker**:				
Yes – No	1.30	0.59	0.51	3.31
**Diabetes**:				
Yes – No	1.14	0.78	0.45	2.88
**Chronic Lung Disease**:				
Yes – No	2.43	0.097	0.85	6.96
**IHD**:				
Yes – No	2.12	0.17	0.73	6.20
**One or more previous repairs**:				
Yes – No	1.67	0.45	0.45	6.22
**Previous Mesh**:				
Yes – No	0.48	0.31	0.12	1.94
**Component Separation**:				
Yes – No	11.8	**< 0.001**	5.79	24.00
**Open**:				
Yes – No	5.64	0.10	0.71	44.6
**IMD Quintile**:				
2 vs. 1	0.43	0.07	0.17	1.06
3 vs. 1	0.29	0.07	0.07	1.10
4 vs. 1	0.42	0.074	0.16	1.09
5 vs. 1	0.9	0.85	0.30	2.67

### Logistic regression – early post operative complications (table 5)

The logistic regression model was statistically significant, χ^2^(16) = 67.1, *p* < 0.001. The model explained 36.5% (Nagelkerke R^2^) of the variance in occurrence of early post operative complications and correctly classified 73.5% of cases. A diagnosis of diabetes was a significant predictor of having an overall early postoperative complication (OR 3.365, *p* = 0.011), as well as having greater than CD 1 wound classification. In terms of deprivation, being in quintile 2 reduced the risk of having postoperative complications significantly when compared to being in quintile 1 (OR 0.0779, *p* = 0.003). There were no other significant predictors of post operative complications.

**Table 5:** Predictive variables for post operative complications (CD1-4) assessed using binomial multivariable logistic regression. P-values in bold are significant (*p* < 0.05)

	Odds Ratio	*P*-value	Lower CI	Upper CI
**Age**	0.99	0.40	0.96	1.02
**Sex**:				
Female – Male	0.89	0.75	0.44	1.81
**BMI**	0.97	0.36	0.91	1.04
**Smoker**:				
Yes – No	1.08	0.89	0.40	2.91
**Diabetes**:				
Yes – No	3.37	**0.01**	1.33	8.55
**IHD**:				
Yes – No	2.11	0.18	0.71	6.25
**Chronic Lung Disease**:				
Yes – No	1.23	0.70	0.43	3.56
**Component Separation**:				
Yes – No	1.73	0.15	0.83	3.62
**Open**:				
yes – no	1.63	0.71	0.126	21.12
**CDC Wound Classification**:				
2–1	7.72	**<0.001**	2.39	24.93
3–1	6.59	**0.001**	2.07	20.95
4–1	7.93	**0.001**	2.25	28.03
**IMD Quintile**:				
2 vs. 1	0.22	**0.003**	0.08	0.61
3 vs. 1	0.29	0.08	0.07	1.17
4 vs. 1	0.68	0.43	0.26	1.77
5 vs. 1	0.89	0.83	0.30	2.60

## Discussion

The outcomes of AWR surgery are dependent on patient factors, technical and systems factors, and the wider social determinants of health [34, 35]. Therefore, improving the patient journey, encompassing prevention, diagnosis, treatment, and rehabilitation, is both a social and medical endeavour. This exploratory study provides insights into patient and surgical factors for 234 patients who have undergone AWR across three hospitals in the North-West region of England. The study’s main findings comprise significantly greater unemployment and smoking rates amongst the most deprived quintiles, and a significant difference in minor operative complications and hospital length of stay between the deprivation groups, despite the groups having similar co-morbidities and baseline hernia characteristics.

Working-age individuals residing in the most deprived areas of England are over twice as likely to be unemployed compared to the national average of 8% [36]. Our study population exhibited greater unemployment rates but a similar pattern of socioeconomic disparity, where Q1 had greater unemployment rates (39.2%) compared to Q5 (19%). This is important in the context of patients undergoing AWR as unemployment has a multifaceted adverse impact on health through economic hardship, psychological stress, and resorting to unhealthy behaviours. Longer periods of unemployment has also been linked to greater disease burden [37]. Similarly, patients from deprived backgrounds are less likely to have formal qualifications and have poorer health literacy [38]. These are all factors which could have contributed to the study findings. Specifically, our results showed that socioeconomic deprivation was associated with greater CD1-2 complications and patients from Q2 had significantly greater risk of prolonged hospital stay compared to Q1. Whilst there are no other studies reporting hernia surgery outcomes by socioeconomic deprivation in the UK, similar findings can be seen from US studies, keeping in mind the differences in healthcare systems – insurance-based (US) versus free at the point of use (NHS). Maskal et al. [19] used the distressed community index (DCI), which is formulated using US Census data and is based on seven indicators of neighbourhood prosperity, including employment. They demonstrated in a database study of over 30,000 patients undergoing ventral hernia surgery that higher DCI correlated with re-admission after surgery, re-operation, and had greater surgical site occurrences [19].

Several retrospective studies from the US have reported disparities in hernia surgery outcomes, using median household income (MHI) as an indicator for socioeconomic status, derived from patient zip codes and US Census Bureau data [15, 20, 39]. In a cohort of 478 patients undergoing complex abdominal wall hernias, Marxen et al. demonstrated a significantly increased risk of overall complications and delayed wound healing amongst patients with low MHI [20]. Bowman et al. reported on 321 patients having ventral hernia surgery having greater likelihood of 30-day readmission [15]. Disparities have also been reported in larger national database studies in patients with low MHI undergoing ventral hernia repairs showing prolonged length of stay, greater risk of inpatient mortality and greater overall complication rates [16–18].

Our findings of greater low-grade complications in the more deprived quintiles could be explained by smoking and diabetes, a known contributory factor to low grade complications such as seromas and wound infections [40]. Within our study population, diabetes was a significant predictor of all grades of post-operative complications, and socioeconomic deprivation was significantly related to having a greater number of current smokers, which is reflective of the disparity seen in large data studies in England [30]. Interestingly, greater rates of prolonged length of stay and CD1-2 complications was observed in the most and least deprived quintiles, which is difficult to explain from the data available.

Understanding the deprivation profile of a patient population is particularly relevant from the standpoint of loco-regional service planning, and crucially, when considering the implementation of a prehabilitation service. There is some evidence that prehabilitation for patients living with obesity leads to reduced risks of complications after abdominal wall reconstruction [41] though the benefits have been better demonstrated in other surgical conditions [42]. Prehabilitation can be integrated within community leisure and health improvement facilities, and it is imperative to identify which localities might experience increased demand to effectively plan and ensure the equitable distribution of resources [43].

Notably, a large proportion (40%) of the patients in this study belonged to the most deprived quintile, and the proportion of patients in each quintile mirrored the distribution of population-level deprivation in the region. Whilst there is no literature to suggest socioeconomic disparities in the prevalence of ventral hernias, there is evidence that some of the risk factors associated with greater hernia occurrence and complications, such as obesity [44] and smoking [45], are more common in more deprived patient groups [30, 46]. It is possible that the prevalence does not significantly vary by deprivation and that the process of selection for an operation in the NHS is non-discriminatory. Alternatively, the true incidence could vary by deprivation, however, as the study only included patients who have undergone elective repair, patients on other pathways will not have been captured. These include patients who have emergency hernia repairs, those on a ‘watch and wait’ pathway, those who are denied surgery altogether (e.g. due to their fitness) and patients who do not present to health services. The difficulty in accurately recording data on these groups of patients, where there is most likely the greatest influence of socioeconomic deprivation, is a challenge in conducting health inequality research in this field. In the UK, a study of Hospital Episode Statistics data reported greater incidence of all types of hernia repairs as an emergency in the most deprived group (23,033 in IMD Q1 vs. 18,614 in Q5) [47]. This could be explained, in part, by greater waiting times in more deprived groups [48], leading to greater rates of obstruction or strangulation [49], necessitating emergency repair.

The IMD is one of the most widely used measures of deprivation in the UK and within its published literature and is helpful in comparing and identifying deprivation profiles of small areas. However, its use is imperfect as it does not accurately identify people’s specific deprivation status, as people from deprived backgrounds may live in non-deprived areas and vice versa. It also doesn’t capture individual factors such as ethnicity and person- specific life experiences contributing to deprivation. Therefore, it could be argued that IMD is a simplified measure of deprivation.

A limitation in the study methodology is having a small sample size compared to larger nationwide studies. Future studies should include patients from a greater geographical area to increase ecological validity. No national hernia database currently exists, and neither would it be possible to link and extract the granular patient and surgical data that this study has reported from NHS England’s Hospital Episode Statistics databases. Future studies should investigate the impact of deprivation and upstream wider determinants of health on the CVH patient journey. This may be best explored through a qualitative study as a quantitative methodology is less suited to capturing intersectionality, the patient’s exposure to experiences which shape their social position and their experiences. It will be possible, as a UK-wide database becomes available, to gain more reliable and generalisable insights into the socioeconomic disparities in AWR surgery.

Over an eight-year period, our study demonstrated some differences in low grade complications and hospital length of stay between the deprivation groups but no difference in high grade complications. It’s possible that the NHS, despite its increasing frailty, has the capacity to avoid the worst complications through equitable care in hospital and equal access to specialist multidisciplinary teams. However, currently, as the NHS faces significant pressures and with growing disparities in healthcare access and health outcomes, the aim must be to optimise NHS functioning to mitigate the impact of socioeconomic deprivation.

## Electronic supplementary material

Below is the link to the electronic supplementary material.

Supplementary Material 1

Supplementary Material 2

Supplementary Material 3

## Data Availability

The data that support the findings of this study are available on request from the corresponding author, Donna Shrestha. The data are not publicly available due to there containing information that could compromise the privacy of research participants.
