# Supplementary material for: Socioeconomic inequalities in patients undergoing abdominal wall reconstruction in the North-West of England, UK: a three-centre retrospective cohort study
Source: Hernia. 2024 Sep 13;28(6):2265–72. doi: 10.1007/s10029-024-03155-0 (PMC11530561; doi:10.1007/s10029-024-03155-0)
Supplement: Supplementary file 1 — Supplementary Material 1 [file 10029_2024_3155_MOESM1_ESM.docx]

**Supplement 1 - Definitions**

**Clavien-Dindo (CD) Score**

| 1 | Any deviation from the normal postoperative course without the need for pharmacological treatment or surgical, endoscopic and radiological interventions.  Acceptable therapeutic regimens are: drugs as antiemetics, antipyretics, analgesics, diuretics, and electrolytes and physiotherapy.  This grade also includes wound infections opened at the bedside |
| --- | --- |
| 2 | Requiring pharmacological treatment with drugs other than such allowed for grade I complications. Blood transfusions, antibiotics and total parenteral nutrition are also included |
| 3 | Requiring surgical, endoscopic, or radiological intervention |
| 3a | Intervention under regional;/local anaesthesia |
| 3b | Intervention under general anaesthesia |
| 4 | Life-threatening complication requiring intensive care/ intensive care unit management |
| 4a | Single organ dysfunction |
| 4b | Multi-organ dysfunction |
| 5 | Patient demise |

**Ventral Hernia Working Group (VHWG) Classification:**

| **Grade 1** | **Grade 2** | **Grade 3** | **Grade 4** |
| --- | --- | --- | --- |
| **Low risk** | **Co-morbid** | **Potentially contaminated** | **Infected** |
| - Low risk of complications  - No history of wound infection | - Diabetes  - COPD  - Immunosuppression  - Active smoker  -Obese | - Previous wound infection  - stoma present  - violation of gastrointestinal tract | - infected mesh  - septic dehiscence |
