# Supplementary material for: Socioeconomic inequalities in patients undergoing abdominal wall reconstruction in the North-West of England, UK: a three-centre retrospective cohort study
Source: Hernia. 2024 Sep 13;28(6):2265–72. doi: 10.1007/s10029-024-03155-0 (PMC11530561; doi:10.1007/s10029-024-03155-0)
Supplement: Supplementary file 2 — Supplementary Material 2 [file 10029_2024_3155_MOESM2_ESM.docx]

**Supplement 2 – Hernia Characteristics**

Frequency and percentage of number of previous repairs (0 or >1). Comparison by IMD quintiles. Percentages displayed are of the overall total or relative to the total number in each IMD quintile, as indicated by n/total. P-values in bold is significant (p<0.05).

|  | Missing  data | Total patient  n (%) | Quintile 1  (Q1) | Quintile 2  (Q2) | Quintile 3  (Q3) | Quintile 4  (Q4) | Quintile 5  (Q5) | P-value |
| --- | --- | --- | --- | --- | --- | --- | --- | --- |
| **First Repair n (%)** | 0 | 149/224(66.5) | 55/88 (62.5) | 35/46 (76.1) | 14/22 (63.6) | 27/40 (67.5) | 18/28  (64.3) | 0.786 |
| **Previous mesh n (%)** | 0 | 64/224 (28.6) | 29/88 (33.0) | 11/46 (23.9) | 5/22 (22.7) | 10/40 (25.0) | 9/28  (32.1) | 0.490 |
| **More than one repair n (%)** | 0 | 80/224 (35.7) | 35/88 (39.8) | 12/46 (26.1) | 8/22 (36.4) | 15/40 (37.5) | 10/28  (35.7) | 0.531 |
| **Hernia Size (CC) (cm)**  **median (IQR)** | 16 | 10.0 (11.0) | 10.0 (13.0) | 10.5 (12.8) | 9.25 (7.50) | 14.3 (13.3) | 11.5  (12.0) | 0.851 |
| **Hernia Size (Trans) cm**  **median (IQR)** | 19 | 10.0 (7.70) | 12.0  (7.0) | 10.0  (9.0) | 8.65 (5.75) | 10.0 (10.5) | 10.0  (7.0) | 0.790 |
| **Ventral Hernia Working Group (VHWG) Classification** | 1 |  |  |  |  |  |  |  |
| **1 n (%)** |  | 44/223 (19.7) | 17/87 (19.5) | 5/46 (10.9) | 3/22 (13.6) | 14/40 (35.0) | 4/28  (14.3) | **<0.001** |
| **2 n (%)** |  | 124/223 (55.6) | 50/87 (57.5) | 27/46 (58.7) | 14/22 (63.6) | 17/40 (42.5) | 16/28  (57.1) | 0.344 |
| **3 n (%)** |  | 35/223 (15.7) | 12/87 (13.8) | 7/46 (12.4) | 4/22 (18.2) | 6/40 (15.0) | 6/28  (21.4) | 0.515 |
| **4 n (%)** |  | 20/223 (9.0) | 8/87  (9.2) | 6/46 (13.0) | 1/22  (4.5) | 3/40  (7.5) | 2/28  (7.1) | 0.312 |
